# Reliability and validity of the cancer-related dysfunctional beliefs and attitudes about sleep scale in cancer patients

**DOI:** 10.1186/s12888-024-05580-y

**Published:** 2024-02-20

**Authors:** Ziyun Gao, Zihan Gao, Chen Zheng, Jianing Ma, Ying Zhao, Lin Zhang, Leilei Guo

**Affiliations:** 1 School of Nursing, Jinzhou Medical University, No.40, Section 3, Songpo Road, Linghe District, Jinzhou City, Liaoning Province P.R. China; 2 Department of Internal Medicine Nursing, School of Nursing, Wannan Medical College, 22 Wenchang West Road, Higher Education Park, Wuhu City, An Hui Province P.R. China; 3 Shengjing Hospital affiliated to China Medical University, Shenyang City, Liaoning Province P.R. China; 4 College of Health Management, Liaoyang Vocational and Technical College, Liaoyang City, Liaoning Province P.R. China

**Keywords:** Insomnia, Cancer patients, Sleep beliefs and attitudes, Reliability, Validity

## Abstract

**Background:**

Insomnia is a common health problem among cancer patients, which is not only a physical problem but also a psychological problem. Sleep plays an important role in the mental and somatic rehabilitation of cancer patients, and the sleep beliefs and attitudes of cancer patients are key factors in improving their sleep situation and quality of life. The aim of this study was to translate the Cancer-Related Dysfunctional Beliefs and Attitudes about Sleep (C-DBAS-14) scale into Chinese and to validate its reliability and validity in cancer patients.

**Method:**

The C-DBAS-14 scale was translated into Chinese using the backward and forward translation procedure. The reliability of the scale was measured by internal consistency, split-half reliability and retest reliability. The validity of the scale was assessed through the content validity indicators, exploratory factor analysis and validation factor analysis.

**Result:**

The Cronbach's ɑ coefficient of the Chinese version of the C-DBAS-14 was 0.932 while the McDonald’s omega coefficient (ω t) was 0.934. The split-half reliability coefficient was 0.908, and the test-retest reliability was 0.857. The four-factor model was obtained using exploratory factor analysis, explaining 72.7% of the variance, with each item loading greater than 0.4 on the common factor. The results of the confirmatory factor analysis revealed that all indicators of model fit were within an acceptable range, indicating a well-fitting model.

**Conclusion:**

The Chinese version of the C-DBAS-14 has good reliability and validity among cancer patients. It can be used to measure the sleep beliefs and attitudes of Chinese cancer patients.

**Supplementary Information:**

The online version contains supplementary material available at 10.1186/s12888-024-05580-y.

## Background

Cancer poses a significant global challenge in the field of public health. The incidence and mortality rates of cancer are increasing rapidly worldwide, seriously jeopardizing human life and health [1, 2]. Cancer is a source of stress, which, together with the effects of anti-tumor therapy, causes a series of physiological and psychological imbalances in patients, with changes in sleep, cognition, and behavior. Sleep disorders, such as insomnia, run through the entire process of diagnosis [3–5], treatment and regression of cancer patients, seriously affecting their quality of life [6, 7]. Several surveys have shown that insomnia is the most common sleep disorder in cancer patients [8–10]. According to several studies, a significant percentage of individuals diagnosed with cancer experience subpar sleep quality, along with challenges in initiating sleep, frequent awakenings, and other sleep-related disorders. The prevalence of these issues ranges from 43 to 80% [11–14], surpassing the rates observed in the general population by a factor of 2 to 3 [15, 16]. There are many factors affecting sleep disorders in cancer patients, which can be divided into three main categories according to a review by Savard and Morin [17]. (1) Susceptibility factors refer to characteristics that predispose an individual to sleep disorders, such as high responsiveness to environmental changes. (2) Predisposing factors or situational conditions of sleep disorders refer to a range of stressors that individuals experience during the diagnosis and treatment of cancer. (3) There are several elements that contribute to the enduring presence of sleep disorders, including false beliefs regarding sleep and maladaptive behaviors related to sleep. Studies have shown that individuals with insomnia or insomnia patients report more cognitive arousal than somatic arousal, that for the causes of poor sleep it is more about the active brain, and that control of active thoughts is more difficult than control of the body [18].

Sleep beliefs refer to an individual's cognition of his or her sleep condition [19]. False sleep beliefs include unreasonable sleep expectations, misperception of the causes of insomnia, exaggerating the adverse effects of insomnia, and attempting to control sleep [20]. Sleep beliefs and attitudes play a rather critical role in the onset, maintenance, and treatment regression of insomnia [21, 22].

False beliefs about sleep can significantly affect the sleep quality, physical condition and mental mood of cancer patients [23–25]. For example, cancer patients tend to obsess over their sleep time, spending too much time in bed or taking naps in order to compensate for insufficient sleep [26, 27]. Daytime naps can have the opposite effect on cancer-related fatigue reduction [28], reducing nighttime sleep quality and total sleep time [23]. This further leads to the occurrence of sleep disorders, adversely affecting the vicious cycle of sleep disorders. The false cognition of sleep in cancer patients creates a serious psychological burden in cancer patients, triggering anxiety, worry, and ultimately non-adaptive behaviors [29, 30]. Excessive concern among cancer patients regarding insufficient sleep is associated with adverse effects on their health. This can exaggerate the negative effects of sleep deprivation and magnify the impact of sleep deprivation on the prognosis of the disease. False beliefs about sleep make cancer insomniacs more prone to thoughts that are so bad that they often hold beliefs such as insomnia may cause long-term negative consequences, which play a prominent role in insomnia's prolongation [31]. Many studies have reported that false sleep beliefs are associated with the occurrence and maintenance of sleep disorders in cancer patients [32, 33]. Therefore, correcting beliefs and attitudes towards sleep in a targeted manner can effectively improve the quality of patients' sleep, alleviate anxiety and depression, help patients adapt to the diagnosis and treatment process of cancer, and increase patients' understanding and control of the disease. This is of great significance in improving the quality of life of cancer patients.

As more and more scholars focus on the importance of sleep beliefs and attitudes in cancer patients, it is particularly vital to find an appropriate tool to assess false sleep perceptions in cancer patients. A widely used tool to assess sleep beliefs and attitudes is the Dysfunctional Beliefs and Attitudes about Sleep scale and a brief version (DBAS-16) [19, 34]. These scales do not distinguish between cancer patients and the general population, nor do they focus on the specific characteristics of cancer patients. Moreover, they have not been validated in the cancer population. Professor Soyoung Youn made revisions to the DBAS scale in 2019, specifically tailored for cancer patients, resulting in the development of the Cancer-Related Dysfunctional Beliefs and Attitude about Sleep scale (C-DBAS-14) [35]. The scale includes two additional items that are pertinent to individuals suffering from cancer. The C-DBAS-14 exhibits specificity and sensitivity towards cancer patients, particularly with their sleep-related cognition. It is capable of distinguishing between cancer patients with insomnia and those with good sleep. Furthermore, it effectively evaluates misconceptions about sleep among cancer patients. The C-DBAS-14 scale has not yet been validated in countries other than South Korea. The primary objective of this study is to assess the reliability and validity of the Chinese version of the C-DBAS-14 among individuals diagnosed with cancer patients.

## Methods

### Design and sample

The aim of this study was to develop and validate a tool for assessing sleep beliefs and attitudes of cancer patients. From November 2022 to May 2023, a questionnaire survey was conducted among cancer patients in two Grade-A tertiary hospitals. The sample size was determined using the general rule for factor analytic procedure with a minimum of 10 respondents for each item [36]. In this study, a total of 356 patients were administered the questionnaire to ensure the accuracy of the experiment. Inclusion criteria: (1) Cancer patients with pathologically confirmed clinical diagnosis; (2) Informed consent and willingness to cooperate with the completion of the study; (3) Clear mind, with good communication skills. (4) Age ≥ 18 years old. Exclusion criteria: (1) the body is too weak to cooperate with the investigator; (2) The expected survival time is less than half a year; (3) Individuals experiencing cognitive impairment or mental illness; (4) Those who have received psychological intervention in the past, which may affect the survey results; (5) People who are unaware of their illness.

### Instruments

#### General demographic characteristics questionnaire

The general situation questionnaire of cancer patients was developed by the researcher, which mainly included age, gender, personal monthly income, marriage, educational level, place of residence, cancer type and cancer stage.

#### The cancer-related dysfunctional beliefs and attitude about sleep

The C-DBAS-14 scale was developed by Prof. Soyoung Youna to measure adverse cognition related to sleep in cancer patients. The scale includes sleep expectations (7 items) and worry about insomnia (2 items), perceived consequences of insomnia and medication (3 items), and two cancer-related items(2 items). There are 14 items in 4 dimensions. Each item was given a Likert-style score ranging from 0 (strongly disagree) to 10 (strongly agree), with higher scores indicating more irrational sleep beliefs. And the Cronbach's α value of the original scale was 0.890 [35].

#### Pittsburgh sleep quality index

The PSQI is a widely used sleep quality survey scale that was developed by Dr. Buysse et al. as a validated measure of subjective sleep quality and sleep disorders over the past month [37]. A higher score on the PSQI is associated with lower sleep quality. The total PSQI score can range from 0 to 21, and a score above 5 indicates subpar sleep quality. The PSQI has demonstrated good reliability (Cronbach’s alpha = 0.83, test–retest reliability = 0.85) [37].

### Procedures

#### Scale translation procedure

We have obtained permission from Professor Soyoung Youn for our translation work. First, two graduate nursing students who were proficient in English independently translated the C-DBAS-14 Scale into two Chinese versions, and then the two translated versions were compared, and the first draft of the Chinese version was formed after consultation and discussion. Two translators who did not know the content of the original scale translated the first draft back into English to form translation version 1 and translation version 2. After completing the translation, the two versions were integrated to form a translation version. The translated scale was basically consistent with the original scale. An expert group meeting was held to compare the translated version with the original version from the perspectives of conceptual equivalence, semantic equivalence, customary equivalence and empirical equivalence, so that the adjusted scale is more in line with the Chinese expression [38, 39]. Item 7”Mood disturbances due to insomnia.” The expert thought that some cancer patients were older and less educated and could not understand "mood disturbance" well, so it was changed to "Low mood is due to insomnia". Item 1 "Need 8 h of sleep," should be changed to "Need 8 h of sleep to stay energized. Item 6 "Better taking sleep pills" was modified to read "It is better to take sleeping pills than to have insomnia". The original scale items 1, 2, 7, 8, 9, 12 and 14 was divided into one dimension named "sleep expectations". However, experts suggested that items 8 and 14 actually measure the patient's concern about insomnia, so it was suggested that items 8 and 14 be combined into the "worry about insomnia" dimension. In addition, experts suggested that the scoring method of the Chinese version of the C-DBAS-14 should be modified so that it is different from the scoring method of the original scale from 0 to 10 [35]. The 10-point Likert scale of the original scale was adjusted to a 5-point Likert scale. Scores from 1 to 5 correspond to (1) Strongly Disagree, (2) Disagree (3) neutral, (4) Agree, and (5) Strongly Agree. Total scores on the scale ranged from 14–70, with higher scores suggesting a greater degree of irrational beliefs. The investigators found that a 5-point Likert scale ranging from strongly disagree to strongly agree provides a more concise and intuitive expression of the patient's beliefs about sleep and is better suited for cancer patients than a 0–10 rating option.

Finally, a convenience sampling method was used to select 30 cancer patients who met the inclusion criteria for the pre-survey in two Grade-A tertiary hospitals. The pre-experiment was used to understand the patients' comprehensibility of the content of the scale and to identify potential problems that may arise. In addition, the scale was further examined for semantic ambiguities and entries that were not easily understood, and timely modifications and adjustments were made.

#### Data collection procedure

After receiving the training, the researchers conducted a questionnaire survey among cancer patients in two Grade-A tertiary hospitals. The researchers distributed paper questionnaires to eligible cancer patients one by one. Before distributing the questionnaires, the researchers provided a comprehensive explanation of the content, purpose, and significance of the study to the patients and their families. The precautions for filling in the questionnaires were told to the patients, and the questionnaires were filled in independently by the patients themselves with the consent of the patients. For patients with low literacy and dysgraphia, the researchers communicated with the patients and filled in the scale on their behalf. The questionnaire was completed and retrieved on the spot, and the investigator checked the quality of the questionnaire on the spot to check whether there were any omissions and misfiled options, and confirmed the modifications with the patients in time to ensure the completeness and quality of the questionnaire. The patients completed the questionnaires within 10–15 min on average, and a total of 356 valid questionnaires were finally collected.

### Statistical analyses

#### Item analysis

The total scores of the 356 cancer sleep belief attitude scale were sorted from high to low, and the values before and after 27% of the total scores of the scale were used as the splitting points of the high and low subgroups, respectively. The independent samples t-test was used to observe the statistical significance of the difference values between the high and low subgroups in the mean number of each entry (*P* < 0.05), and the items with no statistically significant difference were deleted according to the statistical results [40, 41]. Furthermore, this study computed the effect size (Cohen’s d) of the high and low grouping indices. The interpretation of Cohen’s d values was categorized as follows: very small (< 0.20), small (0.20–0.49), medium (0.50–0.79), or large (> 0.80) [42]. The correlation between the score of each item and the total score of the scale was calculated to determine the homogeneity of the scale as a whole. Generally, when the correlation coefficient of the two variables is ≥ 0.4 and reaches the significance level, the homogeneity of the scale is better [38].

#### Validity analysis

The validity of the Chinese version of the C-DBAS-14 includes content validity and structural validity. When the Item-Content Validity Index (I-CVI) ≥ 0.78, the Scale-Content Validity Index (S-CVI) ≥ 0.90, it can be suggested that the content validity of the scale is good [43]. The sample of 356 cases was randomly divided into two groups, one (n = 155) for exploratory factor analysis and the other (n = 201) for validation factor analysis. Exploratory factor analysis was performed by the Weighted Least Squares method with Direct-Oblimin rotation. If the KMO is greater than 0.80 and the Bartlett's test of sphericity is statistically significant, then factor analysis should be conducted [44]. JASP was used for confirmatory factor analysis to analyze whether the fitting index of the model was appropriate. When the chi-square goodness of fit test (CMIN/DF) < 3; Comparative fit index (CFI) > 0.90; the Normed Fit Index (NFI) > 0.85; Goodness of Fit Index (GFI) > 0.90, the model can be considered to be a full fit to the data [45, 46]. In order to evaluate the construct validity of the item measures, we also examined the convergent validity and discriminant validity.

#### Reliability analysis

The internal consistency of the translated scale and each dimension was assessed using Cronbach's α coefficients and McDonald’s omega coefficient. The value of 0.7 is regarded as satisfactory for internal consistency [47]. Additionally, the stability and consistency of the scale over time were examined by retesting 30 cancer patients after a two-week interval, utilizing a post-translation scale. Split-half reliability was determined by dividing the scale items into two halves and calculating the correlation between the results of these two halves.

#### Ethics committee

All individuals have provided informed consent before the data collection.

## Results

### The sample

The demographic characteristics of the participants involved in this study are outlined in Table 1. The average age of the patients was 60.16 years, with the majority of participants being male, accounting for 52.5%. Furthermore, a significant proportion of the participants were married (92.7%). In terms of clinical background, the majority of patients diagnosed with lung cancer accounted for 28.4%, while 16.6% were diagnosed with breast cancer. The highest proportion of patients, 41.6%, were in stage 3 of cancer. Among them, 69.7% of cancer patients who had a PSQI score greater than 5 were diagnosed with poor sleep quality.

**Table 1:** Frequency distribution of demographic characteristics (*n* = 356)

**Variables**	Groups	*N*	**%**
**Gender**	Male	169	47.5
	Female	187	52.5
**Age(years)**	≤ 35	7	2.0
	35–44	34	9.6
	45–54	72	20.2
	55 ~ 64	127	35.7
	65 ~ 74	98	27.5
	75 ~ 84	16	4.5
	≥ 85	2	0.6
**Educational levels**	Primary school or less	63	17.7
	Junior high school	146	41.0
	Senior high school	81	22.8
	Junior college	42	11.8
	Bachelor degree or above	24	6.7
**Place of residence**	City	209	58.7
	Countryside	147	41.3
**Personal monthly income**	≤ 1000	53	14.9
	1000 ~ 3000	130	36.5
	3000 ~ 5000	108	30.3
	≥ 5000	65	18.3
**Cancer stages**	Stage I	15	4.2
	Stage II	68	19.1
	Stage III	148	41.6
	Stage IV	125	35.1

### Item analysis

The critical ratio (CR) values for the 14 items of the scale ranged from 13.85 to 23.91, and the Cohen's d values ranged from 1.95 to 3.37, all of which were greater than 0.8. The score of each item was positively correlated with the total score of the scale (*r* = 0.643 to 0.789, *P* < 0.001). After deleting each item, the Cronbach's ɑ values for the scale ranged from 0.925 to 0.930; the McDonald's omega values were 0.927–0.931, which did not exceed the original Cronbach's α values and McDonald's omega values. After item analysis, no items were deleted (Table 2 and Table supplement 1).

**Table 2:** Discriminant validity analysis of the Chinese version of the scale (*n* = 356)

**Item**	**Low-score group mean** ± ***SD***	**High-score group mean** ± ***SD***	***t***	***P***	***Cohen’s d***
Q1	2.10 ± 0.47	4.02 ± 0.65	23.91	< 0.001	3.37
Q2	2.05 ± 0.64	4.06 ± 0.87	18.72	< 0.001	2.62
Q3	1.68 ± 0.80	4.05 ± 0.88	19.82	< 0.001	2.81
Q4	1.69 ± 0.77	3.97 ± 0.83	20.13	< 0.001	2.84
Q6	1.12 ± 0.33	3.49 ± 1.08	21.14	< 0.001	2.94
Q7	2.29 ± 0.66	4.10 ± 0.70	18.78	< 0.001	2.66
Q8	2.05 ± 0.68	3.71 ± 0.84	15.29	< 0.001	2.17
Q9	2.13 ± 0.67	4.05 ± 0.67	20.20	< 0.001	2.87
Q12	2.44 ± 0.74	4.23 ± 0.72	17.32	< 0.001	2.45
Q13	1.33 ± 0.70	3.64 ± 0.82	21.37	< 0.001	3.02
Q14	1.99 ± 0.64	3.83 ± 0.75	18.71	< 0.001	2.63
Q15	1.20 ± 0.40	3.48 ± 1.12	19.43	< 0.001	2.68
Q17	2.53 ± 0.81	4.00 ± 0.70	13.85	< 0.001	1.95
Q18	2.53 ± 0.71	4.08 ± 0.74	15.10	< 0.001	2.14

### Validity analysis

#### Content validity

The Chinese version of the C-DBAS-14 was assessed by a panel of seven experts. The seven experts cover three specialties: clinical oncology medicine, clinical oncology nursing and psychology. The I-CVI ranged from 0.857 to 1.000 and the S-CVI was calculated to be 0.949.

### Construct validity

#### Exploratory factor analysis

**Fig. 1:**
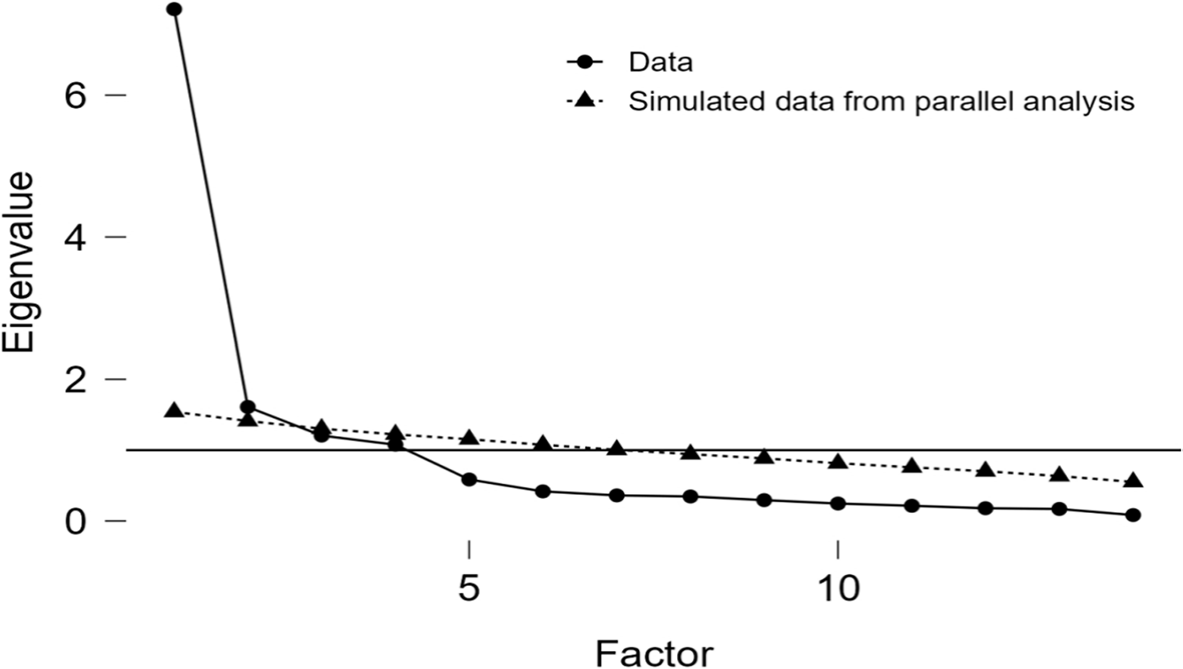
Screen plot of exploratory factor analysis for Chinese version of the C-DBAS-14

The adequacy of sampling is supported by KMO = 0.87. It measures the quality of sampling and the quality of the correlation matrix through Bartlett's test of significance (*χ2* = 1623.907, *p* < 0.001). The results indicate that four factors were extracted, which accounted for 72.7% of the variance. Factor 1 accounted for 25.8% of the variance (eigenvalue = 7.21), factor 2 accounted for 16.4% (eigenvalue = 1.21), factor 3 accounted for 16.7% (eigenvalue = 1.61) and factor 4 accounted for 13.8% (eigenvalue = 1.08). A 4-factor model was also corroborated by the results of the scree plot (Fig. 1). The results show that the structure and item attribution of the Chinese C-DBAS-14 are basically consistent with the original scale, and each factor load ranges from 0.463 to 0.969 (Table 3).

**Table 3:** Factor load and uniqueness of each item in C-DBAS-14 of 14 Items (*n* = 155)

**Item**	**F1**	**F3**	**F2**	**F4**	**Uniqueness**
D7	**0.855**				0.264
D2	**0.812**				0.322
D1	**0.796**				0.240
D9	**0.749**				0.351
D12	**0.674**				0.424
D15		**0.846**			0.256
D6		**0.818**			0.193
D13		**0.803**			0.249
D4			**0.934**		0.100
D3			**0.740**		0.239
D8			**0.488**		0.532
D14			**0.463**		0.412
D17				**0.969**	0.065
D18				**0.837**	0.175

F1 contained Q1, Q2, Q7, Q9, and Q 12, F2 contained Q3, Q4,Q8 and Q14, F3 contained Q6, Q13 and Q15, F4 contained Q17and Q18

### Confirmatory factor analysis

**Fig. 2:**
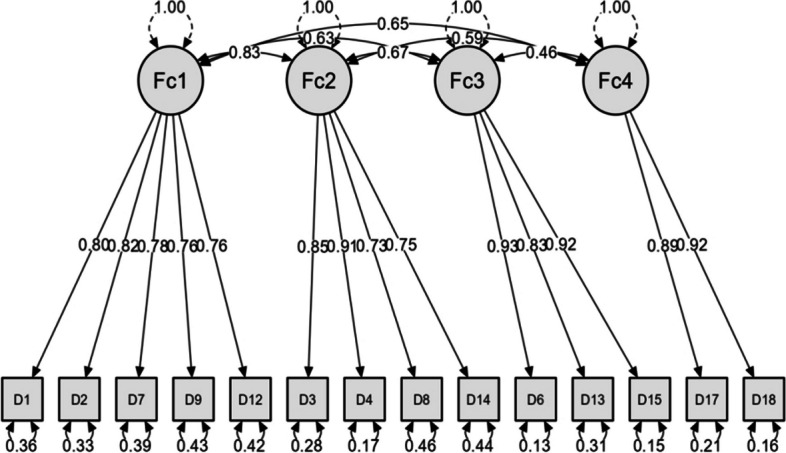
Standardized four-factor structural model of the Chinese version of the C-DBAS-14 (*n* = 201)

The CFA results provided evidence for the four-factor structure of C-DBAS-14. The model fit indices CMIN/DF = 2.255, NFI = 0.927 and RMSEA = 0.079, which are in the acceptable range. This is shown in Table 4 and Fig. 2. All fitting indices demonstrate a good fit of the model used in this study with the data utilized.

**Table 4:** Evaluation fitness of C-DBAS-14 model

**Model**	**CMIN/DF**	**NFI**	**RFI**	**IFI**	**TLI**	**CFI**	**PNFI**	**RMSEA**
Final model	2.255	0.927	0.906	0.958	0.946	0.957	0.723	0.079
Standard value	< 5.000	> 0.900	> 0.900	> 0.900	> 0.900	> 0.900	> 0.500	< 0.08

Through the analysis of the correlation coefficient between the partial scale and the whole scale, it is found that the $\sqrt{AVE}$ value of each subscale is between 0.79 and 0.91, which is greater than the correlation coefficient of the two indicators, indicating that there are significant differences between each subscale in this study, and the internal structure of the questionnaire is highly differentiated. (The results are shown in Table 5) The average variance extracted (AVE) values of the four subscales ranged from 0.62 to 0.82, and the construct reliability values (CR) ranged from 0.89 to 0.92 (AVE > 0.5; CR > 0.7), indicating that the questionnaire compiled in this study has good convergence validity.

**Table 5:** Results of confirmatory factor analysis

Factor	Item	Parameter significance estimation					Correlation between factors (Discriminant Validity)				Construct Reliability	Convergent Validity	
		Unstd. Estimate	S.E	C.R	*P*	Std. Estimate	F1	F2	F3	F4	CR	AVE	$\sqrt{AVE}$
F1	D1	1.00			< 0.001	0.80	1				0.89	0.62	0.79
	D2	1.10	0.09	12.99	< 0.001	0.82							
	D7	0.95	0.08	11.99	< 0.001	0.78							
	D9	0.96	0.08	11.56	< 0.001	0.76							
	D12	0.94	0.08	11.69	< 0.001	0.76							
F2	D3	1.00			< 0.001	0.85	0.762	1			0.89	0.66	0.81
	D4	1.07	0.06	17.08	< 0.001	0.91							
	D8	0.69	0.06	11.70	< 0.001	0.73							
	D14	0.68	0.06	11.97	< 0.001	0.75							
F3	D6	1.00			< 0.001	0.93	0.581	0.617	1		0.92	0.80	0.89
	D13	0.90	0.05	16.82	< 0.001	0.83							
	D15	0.99	0.05	21.25	< 0.001	0.92							
F4	D17	1.00			< 0.001	0.89	0.581	0.548	0.444	1	0.90	0.82	0.91
	D18	1.00	0.08	13.35	< 0.001	0.92							

### Reliability analysis

The Cronbach's α value for the Chinese version of the C-DBAS-14 was found to be 0.932, while the McDonald's omega value was 0.934. Furthermore, each dimension of the scale exhibited satisfactory reliability, with Cronbach's α value ranging from 0.877 to 0.914 and McDonald omega values ranging from 0.81 to 0.914. The split-half reliability coefficient was calculated to be 0.908, suggesting reliable measurement. For the purpose of retesting, a sample of thirty participants was selected, and the retest reliability coefficient was determined to be 0.857 after a two-week interval.

## Discussion

The data shows that in 2020, the number of newly diagnosed cancer cases in China exceeded 4.56 million, accounting for 23.7% of the global total of newly diagnosed cancer cases [48]. The prevalence of sleep disorders, including insomnia, is relatively high among cancer patients, which has a detrimental impact on their overall quality of life and cancer treatment outcomes [49–51]. Sleep beliefs and attitude play a crucial role in the onset, development and maintenance of insomnia problems, as well as in their treatment. Tools widely used in China to assess sleep cognition have not been validated in cancer patients and are difficult to accurately respond to false sleep beliefs in cancer patients. The C-DBAS-14 developed by Prof. Sunyong fills this gap by accurately identifying cancer patient characteristics and is particularly sensitive to cancer patients, reflecting the beliefs of sleep disorders specific to cancer patients [35]. Studies have shown that sleep deprivation has been found to lead to altered immune function, which can result in decreased levels of immunoglobulin, complement, and some cellular subpopulations, leading to impaired immune function and exacerbation of the patient's condition [52–54]. Compared to the general population, cancer patients exhibit heightened concern regarding the detrimental effects of sleep deprivation on immune function and cancer recovery. They perceive this matter as a matter of life and death. In this particular scenario, cancer patients often develop erroneous beliefs regarding the crucial role of sleep at specific times in maintaining immune function, as well as the serious detrimental impact of poor sleep on cancer progression [31, 55, 56]. The two new items in the C-DBAS-14 do a good job of covering false beliefs about sleep that are unique to cancer patients and are an effective tool for assessing sleep beliefs and attitudes in cancer patients.

The results show that the Chinese version of C-DBAS-14 has the appropriate reliability and validity to evaluate incorrect beliefs about sleep in cancer patients. It provides a theoretical basis for clinicians and nurses to more accurately and efficiently describe poor sleep cognition in cancer patients, identify it in time, and develop intervention strategies. And this instrument serves as a decision support for clinical research and nursing practice, with the aim of enhancing the sleep quality of cancer patients.

In this study, the C-DBAS-14 scale was translated, translated back, and culturally adjusted according to the Brislin translation model, and the Chinese version of the C-DBAS-14 was formed through pre-investigation and expert consultation, which included 14 items in 4 dimensions. The division of 14 items in this study deviates slightly from the original scale dimensions. Due to the background and cultural habits in China, the semantics of item 8 and item 14 place a stronger emphasis on the concerns of cancer patients regarding the consequences of insomnia. The adjusted structure of the scale is now more clear and explicit. The item analysis of the project indicates that the Chinese version of the C-DBAS-14 demonstrates excellent discrimination. Additionally, Cronbach's α values and McDonald’s omega values after the removal of each item do not exceed the original values of the translated scale.

This study conducted a reliability valuation of the Chinese version of the C-DBAS-14 from three aspects: internal consistency reliability, test–retest reliability, and split-half reliability. The results showed that the Cronbach's α value of the scale was 0.932 and the McDonald omega value was 0.934, which was higher than the results of the Korean version [14]. The split-half reliability and test–retest reliability were both within an acceptable range, indicating that the scale has a high level of stability and can be used repeatedly in the cancer population. The Chinese version of the C-DBAS-14 exhibits desirable reliability in the cancer population.

The validity of a scale consists of content validity and structural validity. The Chinese version of the C-DBAS-14 demonstrated an acceptable I-CVI range of 0.857 to 1.000, with an S-CVI of 0.949, which exceeded the reference values and met the relevant requirements. Structural validity is an important indicator to evaluate the stability of the scale structure, commonly assessed through exploratory factor analysis. In general, it is believed that exploratory factor analysis should extract common factors that align with the original scale, and the cumulative variance contribution should be above 40% [57]. In the original scale, three common factors with eigenvalues greater than 1 are extracted, and the goodness of fit of each index of the four-factor model is found to be good by comparing the fitting index. Finally, the four-factor structure is adopted [35]. In this study, four common factors were extracted with a cumulative variance contribution of 72.7%, which is consistent with the common factors of the original scale. This study further verified the structure of the Chinese version of C-DBAS-14 using confirmatory factor analysis. The model fit indices showed that all of them reached the ideal fitting standard, indicating that the scale structure was stable. The construct reliability (CR) ranged from 0.89 to 0.92, higher than the reference value of 0.70, demonstrating convergent validity. Additionally, the fact that the square of the correlation coefficients between latent variables was smaller than the average variance extracted value also confirmed discriminant validity. Overall, the Chinese version of C-DBAS-14 showed high validity among cancer patients.

### Limitation and strength

The limitations of this study lie in its cross-sectional design and the fact that the participants were all from one province. In the future, it is necessary to expand the sample size and conduct a multi-center longitudinal investigation in more provinces and hospitals to investigate the perception and beliefs of cancer patients regarding sleep from a dynamic perspective.

This study, for the first time, translated the C-DBAS-14 scale into Chinese and validated its reliability and validity among cancer patients, providing an effective and accurate tool for assessing erroneous cognition in cancer patients in the future.

## Conclusion

The Chinese version of the C-DBAS-14 has sound psychometric properties. The Chinese version of the C-DBAS-14 is sensitive to cancer patients, has a clear and simple structure, and can be used to measure the sleep beliefs and attitudes of cancer patients. The scale provides a theoretical basis for healthcare professionals to scientifically manage the sleep-related cognition of cancer patients, which is of significant importance in enhancing the quality of life for cancer patients.

### Supplementary Information

**Additional file 1.** C-DBAS-14 item-total score Pearson correlation analysis results (*n*=356, α=0.05).

## Data Availability

The datasets generated and/or analyzed during the current study are not publicly available to preserve anonymity of the respondents but are available from the corresponding author on reasonable request.
