# Supplementary material for: Reliability and validity of the cancer-related dysfunctional beliefs and attitudes about sleep scale in cancer patients
Source: BMC Psychiatry. 2024 Feb 20;24:144. doi: 10.1186/s12888-024-05580-y (PMC10880202; doi:10.1186/s12888-024-05580-y)
Supplement: Supplementary file 1 — Additional file 1. C-DBAS-14 item-total score Pearson correlation analysis results (n=356, α=0.05). [file 12888_2024_5580_MOESM1_ESM.docx]

**C-DBAS-14 item-total score Pearson correlation analysis results（n=356, α=0.05）**

| **Item** | **Item content** | ***r*** | ***P*** |
| --- | --- | --- | --- |
| **Q1** | Need 8 hours of sleep | 0.773 | <0.001 |
| **Q2** | Need to catch up on sleep loss | 0.743 | <0.001 |
| **Q3** | Consequences of insomnia on health | 0.789 | <0.001 |
| **Q4** | Fear of losing control over sleep | 0.777 | <0.001 |
| **Q6** | Better taking sleeping pills | 0.756 | <0.001 |
| **Q7** | Mood disturbances due to insomnia | 0.744 | <0.001 |
| **Q8** | One poor night disturbs whole week | 0.692 | <0.001 |
| **Q9** | Cannot function without a good night | 0.752 | <0.001 |
| **Q12** | Lack of energy due to poor sleep | 0.709 | <0.001 |
| **Q13** | insomnia resulting from chemical imbalance | 0.731 | <0.001 |
| **Q14** | Insomnia destroying life | 0.749 | <0.001 |
| **Q15** | Medication as a solution | 0.724 | <0.001 |
| **Q17** | Problems with immune system without sleep | 0.643 | <0.001 |
| **Q18** | Cancer may recur or metastasize | 0.659 | <0.001 |
